# Overview: does language production shape language form and comprehension?

**DOI:** 10.3389/fpsyg.2013.00458

**Published:** 2013-07-18

**Authors:** Charles Clifton, Manuel Carreiras

**Affiliations:** ^1^Department of Psychology, University of Massachusetts AmherstAmherst MA, USA; ^2^Basque Center on Cognition, Brain and LanguageSan Sebastián, Spain; ^3^IKERBASQUE. Basque Foundation for ScienceSan Sebastián, Spain

Analyzing how predictability, especially statistically-based predictability, affects language comprehension has become a major theme in psycholinguistics (e.g., Jurafsky, 1996; Hale, 2001; Lau et al., 2006; Staub and Clifton, 2006; Levy, 2008). This interest can be traced back to a series of papers (notably MacDonald et al., 1994) challenging the received claim that frequency is largely irrelevant to language, combined with observations that young language learners are very sensitive to statistical regularities (e.g., Saffran et al., 1996). It is now clear that adult language users comprehend frequent words, phrases, constructions, etc. more readily than less frequent ones. What is not so clear is the nature of the relation between frequency and comprehensibility. Is their relation superficial, perhaps reflecting the action of other causal factors? Is the relation causal, but mediated by processes other than a direct mapping of experience onto expectation? What gets counted in determining frequency? Is frequency just one of many factors that affect comprehension, or is it the dominant one? Whatever its nature, why does the relation between frequency and comprehension exist?

In the core target article of this Topic, Maryellen MacDonald assumes that there is a direct and immediate relation between frequency of experience and comprehensibility, and asks, what is the reason for the frequency differences? She advances the Production-Distribution-Comprehension (PDC) approach, which claims that a speaker/writer has biases that reduce production difficulty. These biases result in some linguistic forms being more common than others, and listener/readers learn to take advantage of the resulting regularities. She lays out three biases, which one commentator humorously terms “blurt, mimic, space,” and shows how they can account for some phenomena of sentence comprehension and language typology.

The Research Topic contains commentaries from a bakers dozen (and more) prominent psycholinguistic researchers. They uniformly applaud MacDonald's program, recognizing that an account of statistical regularities is needed and finding promise in basing the regularities on production constraints. But given that they are academic researchers, each commentator necessarily finds some shortcomings in MacDonald's proposal. Some note that the approach described in her paper is an approach, not an explicit, implemented, testable theory. Some suggest that there are grammatical phenomena, and grammatical differences among languages, that are not plausibly based in simple applications of the production biases MacDonald proposes. Several argue that considerations of the perceiver must play a larger role than MacDonald gives them, and some argue that an integrated account of production and comprehension demands is needed. In a related vein, some commentators argue that information structure and communication pressures play more of a role in statistical regularities than the PDC approach provides. Finally, some commentators argue for a broadening of the evidential base and even the underlying theoretical assumptions. They argue that language is not produced or comprehended in isolation, and suggest that we should consider communication not as the transmission of a message from source to receiver, but as the reduction of mutual uncertainty.

Frontiers' Research Topics format permitted this lively exchange of proposal and critiques. In her rejoinder, MacDonald acknowledges some of the current shortcomings of her approach, but also argues that some have been overcome in her other work and that others are well worth addressing. The availability of the discussion in this Research Topic will certainly stimulate researchers to evaluate the PDC proposal and to formulate alternatives, and thus advance our understanding of the nature of language.

## References

[B1] Hale J. (2001). A probabilistic earley parser as a psycholinguistic model. Proc. NAACL 2, 156–166

[B2] Jurafsky D. (1996). A probabilistic model of lexical and syntactic access and disambiguation. Cogn. Sci. 20, 137–194 10.1207/s15516709cog2002_1

[B3] Lau E. Straud C. Plesch S. Phillips C. (2006). The role of structural prediction in rapid syntactic analysis. Brain Lang. 98, 74–88 10.1016/j.bandl.2006.02.003 16620944

[B4] Levy R. (2008). Expectation-based syntactic comprehension. Cognition 106, 1126–1177 10.1016/j.cognition.2007.05.006 17662975

[B5] MacDonald M. C. Pearlmutter N. J. Seidenberg M. S. (1994). The lexical nature of syntactic ambiguity resolution. Pyschol. Rev. 101, 676–703 10.1037/0033-295X.101.4.676 7984711

[B6] Saffran J. R. Aslin R. N. Newport E. L. (1996). Statistical learn-ing by 8-month-old infants. Science 274, 1926–1928 10.1126/science.274.5294.1926 8943209

[B7] Staub A. Clifton C. J. (2006). Syntactic prediction in language comprehension: Evidence from either ….or. J. Exp. Psychol. Learn. Mem. Cogn. 32, 425–436 10.1037/0278-7393.32.2.425 16569157 PMC1479855

